# Venous Tropism in Renal Cell Carcinoma: A Rare Imaging Presentation

**DOI:** 10.7759/cureus.70927

**Published:** 2024-10-06

**Authors:** Nadeem ur Rahman, Hemanth Neeli, Ayush Gupta, Jitendra Sharma, Pratik Minj

**Affiliations:** 1 Radiology, All India Institute of Medical Sciences, Bhopal, Bhopal, IND; 2 Pathology, All India Institute of Medical Sciences, Bhopal, Bhopal, IND

**Keywords:** cect - contrast enhanced computed tomography, ivc thrombus, renal cell carcinoma (rcc), ultra sound, venous tumor thrombus

## Abstract

Renal cell carcinoma (RCC) is one of the most prevalent malignancies in adults. Accurate staging and assessment of tumor extent are critical for effective management. RCC often demonstrates a tendency for venous invasion, commonly extending into the renal vein and inferior vena cava. However, RCC extending into the gonadal veins is an uncommon manifestation. In this report, we present the imaging features of a 62-year-old woman presented with left flank pain. The ultrasound examination revealed a large hyperechoic mass in the left renal fossa, replacing almost the entire left kidney with the contiguous extension of the tumor into the left renal vein and ovarian vein. Color Doppler showed the presence of flow within the thrombus, confirming the presence of a malignant thrombus. Contrast-enhanced CT examination revealed a large heterogeneously enhancing mass in the left kidney with the contiguous extension of tumor thrombus in the left renal vein, IVC, and into the left ovarian vein. This report highlights how imaging techniques can be instrumental in detecting and characterizing rare but significant tumor extensions, which can have crucial prognostic implications for overall patient management.

## Introduction

Renal cell carcinoma (RCC) is a common and aggressive form of renal neoplasm, representing a significant health burden among adults [1]. Over 50% of RCCs are detected incidentally, making the classic triad of flank pain, gross hematuria, and palpable mass less common. Clinically suspected RCC are initially evaluated with laboratory tests such as serum creatinine, hemoglobin, leukocyte counts, urine analysis, and C-reactive protein, many of which are used for prognosis. Imaging, particularly ultrasound and CT scans, is central to diagnosis, with MRI useful for assessing venous involvement and local infiltration of the adjacent soft tissues. Contrast-enhanced chest, abdominal, and pelvic CT is essential for staging. Renal core biopsy confirms the histopathological diagnosis and is especially recommended before ablative therapies and in patients with metastasis before commencing systemic treatment [2]. Effective management of RCC heavily relies on accurate staging and comprehensive assessment of tumor spread, particularly given its propensity for venous invasion [3,4]. While RCC frequently extends into the renal vein and inferior vena cava (IVC), involvement of the gonadal veins is rare [5]. Through the use of ultrasound, color Doppler, and contrast-enhanced CT imaging, this case illustrates the critical role of advanced imaging techniques in diagnosing and assessing atypical tumor spread, which can significantly influence treatment decisions and patient outcomes.

## Case presentation

A 62-year-old woman presented to the urology department with a one-week history of macroscopic hematuria and severe left flank pain. The general examination was unremarkable except for mild tachycardia. No known bleeding diathesis requiring anticoagulant medication were found. She was a chronic diabetic and poorly compliant with oral medicines. She was a non-smoker and had no family history of cancer. Abdominal examination revealed a large, vague, ill-defined lump in the left lumbar region. No tenderness or signs of peritoneal irritation were noted. Laboratory tests showed decreased red blood cell count (3.5 million/mm^3^), reduced hemoglobin levels (8 g/dl), and mild renal insufficiency (creatinine 1.3 mg/dL).

**Figure 1:**
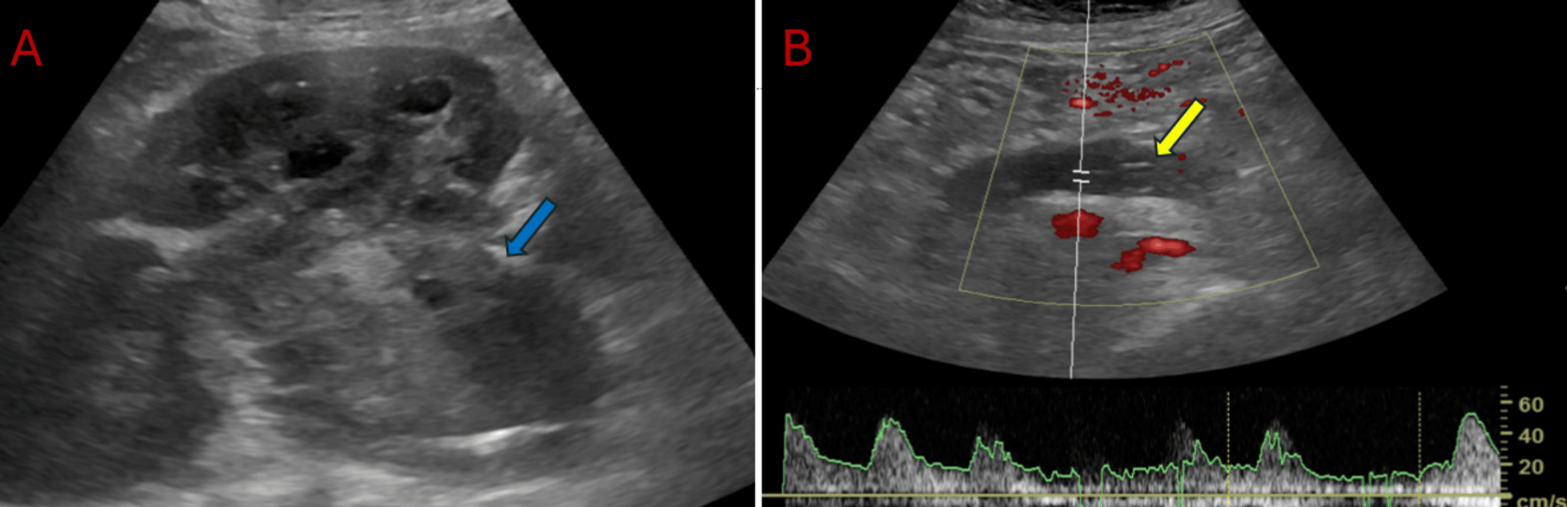
Ultrasound imaging of kidney with Doppler study Ultrasonography images with B mode (A) showing a large exophytic lobulated heteroechoic mass arising from the kidney (blue arrow) and on Power Doppler imaging with spectral waveforms. (B) reveals hypoechoic mass within the left gonadal vein, expanding it with the presence of intermixed arterial and venous waveforms suggesting tumor thrombus (yellow arrow).

**Figure 2:**
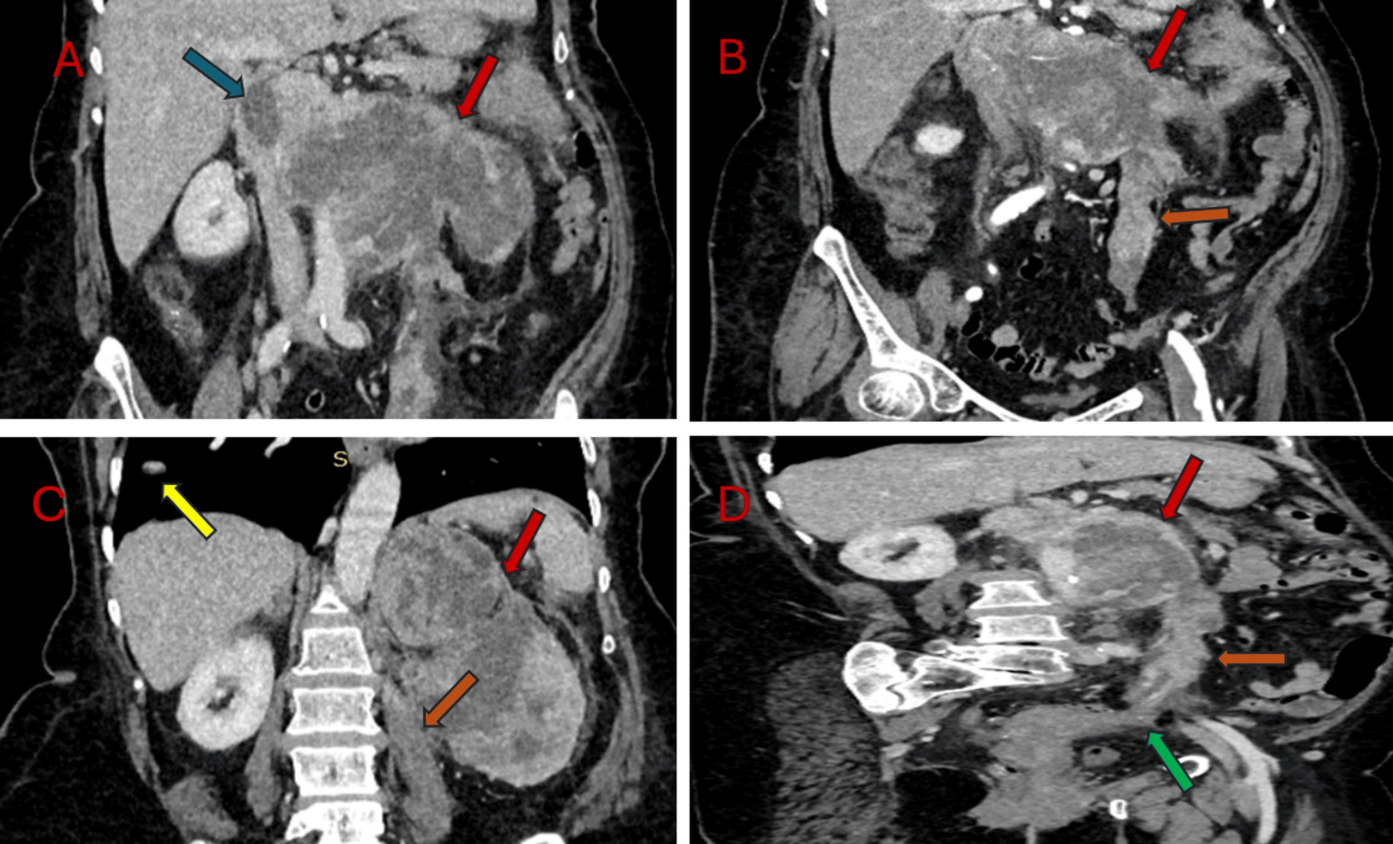
Contrast enhanced tomography images depicting the renal mass and its locoregional extension (A) Coronal image showing heterogeneously enhancing renal mass (red arrow) with invasion of the ipsilateral renal vein and IVC (blue arrow); (B) Coronal image showing continuous extension of tumor thrombus into left ovarian vein (orange arrow); (C) Coronal image showing pulmonary metastasis (yellow arrow) with a large left renal mass (red arrow); (D) Oblique reformatted coronal image showing continuous extension of tumor thrombus with focal infiltration into ipsilateral parametrium and fallopian tube (green arrow). IVC: inferior vena cava

An initial abdominal ultrasound examination revealed a large mass of heterogeneous echogenicity occupying the left renal fossa, nearly replacing the entire left kidney with contiguous extension and expansion of the left renal vein and IVC (Figure 1A). On the color Doppler, it showed internal areas of arterial flow, suggesting a tumor thrombus (Figure 1B). A CECT scan of the chest, abdomen, and pelvis was performed for further evaluation and staging. A CT urography protocol with an IV dose of iodinated contrast, comprising corticomedullary, nephrographic, and excretory phase scans, was obtained. It revealed a large heterogeneously enhancing mass measuring approximately 9.5 x 16.2 x 15.5 cm (AP x TR x CC) in maximum orthogonal dimensions, replacing most of the renal parenchyma with associated mild perinephric fat stranding (Figure 2A). The mass contiguously extended into the left renal vein, IVC up to the distal intrahepatic segment, and retrogradely into the left ovarian vein, ~15 cm, with infiltration of the left parametrium, left lateral wall of the uterine fundus, and left fallopian tube (Figures 2B, 2D). Curved multiplanar reformat through the IVC and right ovarian vein confirmed these findings and accurately assessed the craniocaudal extension of venous malignant invasion (Figure 2D). The tumor thrombus expanded the involved veins and showed arterial neovascularity and heterogeneous contrast enhancement on the venous phase, similar to the primary mass. Along with multiple bilateral pulmonary metastases (Figure 2C), imaging findings are consistent with extensive metastatic RCC.

**Figure 3:**
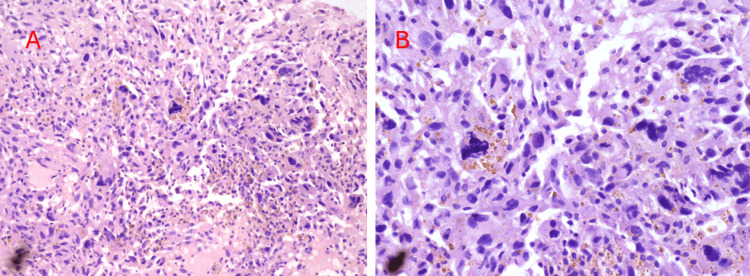
Photomicrographs depicting tumor histology (A) Tumor cells show moderate pleomorphism, hyperchromatic nuclei, irregular nuclear membrane, and moderate to scant cytoplasm; (B) Bizarre tumor cells with the presence of mitotic figures.

Given the substantial burden of primary and metastatic disease and the invasion of the venous structures, the patient was considered an unsuitable candidate for primary resection. Hence, a percutaneous CT-guided biopsy was done, which revealed multiple pleomorphic tumor cells arranged in sheets and showed high mitotic figures corresponding to poorly differentiated malignant RCC (Figures 3A, 3B). Due to the extensive local extension of the tumor, consistent with stage T4A or IVA, according to the TNM and Robson classifications, respectively. The patient elected to undergo immunotherapy with sunitinib. The patient tolerated the chemotherapy well and planned for imaging follow-up for the treatment response.

## Discussion

RCC makes up about 90% of all renal cancers and roughly 2% of all adult malignancies [1]. Accurate preoperative tumor staging is mandatory for estimating disease burden, vascular invasion, presence of metastasis, and resectability and for optimal surgical planning. RCC is notorious for invasion of the venous structures, a phenomenon called venous tropism [3]. It usually invades the renal vein and can extend up to the right atrium through the IVC. Ovarian vein tumor invasion is a relatively rare occurrence, and to the best of our knowledge, less than 15 cases have been reported in the medical literature [5]. Our case is unique owing to the direct contiguous infiltration of the ipsilateral fallopian tube and uterus, occurring along the pathway of the ovarian vein. Only one case was reported by Sweigert et al. and has shown findings similar to ours [6]. Despite its rarity, it is essential to look for this finding since non-recognition can lead to residual disease post-surgery, implying poor patient survival.

Preoperative imaging utilizing multi-detector computed tomography (MDCT) is instrumental in staging and assessing the disease's burden. It provides comprehensive preoperative staging and information such as vascular anatomy and vascular collateral formation, which can mitigate perioperative complications and guide optimal surgical management. Complementary imaging techniques, such as Doppler ultrasound, are valuable for differentiating between bland thrombus and tumor thrombus.

Ovarian veins originate from the venous plexus in the parametrium near the adnexa, communicate with the uterine plexus, and traverse anterior to the ureter and psoas muscle. The left ovarian vein drains into the left renal vein, providing a potential pathway for retrograde extension of a left renal mass into the left ovarian vein. This can lead to contiguous invasion of the local adnexal structures. Although direct tumor extension along this pathway is less commonly observed than metastasis to the fallopian tubes or ovaries, the renal-ovarian axis [6,7] is recognized as a possible route for such metastatic spread.

A precise assessment of ovarian vein involvement is crucial when planning an aggressive surgical approach with curative intent. On multidetector computed tomography (MDCT), ovarian vein invasion is indicated by a heterogeneously enhancing soft tissue mass during the nephrographic phase of contrast enhancement, signifying a malignant thrombus due to tumor invasion [8,9]. Conversely, a bland thrombus, resulting from extrinsic compression or coagulation dysfunction, will appear as a non-enhancing, static intraluminal mass on MDCT. Power Doppler ultrasound provides additional diagnostic information by revealing a distended echogenic thrombus and demonstrating both arterial and venous flow patterns. This helps differentiate a malignant thrombus, characterized by abnormal or increased flow, from a bland thrombus with minimal or no flow. Thus, MDCT and Doppler ultrasound offer crucial information for distinguishing between malignant and benign thrombus conditions, which is vital for accurate staging and optimal treatment planning [10].

This case report underscores the significance of identifying ovarian vein invasion by RCC through the use of dedicated MDCT and power Doppler ultrasound examinations. Thus, it is essential for urologists and radiologists to be vigilant for this potential finding during RCC staging and to incorporate the evaluation of the ovarian veins into their diagnostic protocol. Such cases should be managed with a surgical strategy akin to that used for renal vein and IVC invasion, which may necessitate an aggressive surgical approach, including ipsilateral oophorectomy.

## Conclusions

In conclusion, this case underscores the importance of thorough preoperative imaging and vigilance in detecting rare venous extensions of RCC, such as ovarian vein invasion. Although uncommon, direct tumor spread via the ovarian vein can have significant implications for surgical planning and patient prognosis. The use of advanced imaging modalities like MDCT and power Doppler ultrasound proves invaluable for distinguishing between benign and malignant thrombus, guiding the surgical approach, and preventing residual disease. By incorporating ovarian vein evaluation into routine diagnostic protocols for RCC, clinicians can improve the detection of atypical tumor spread and optimize patient outcomes with more targeted, aggressive surgical strategies.
